# Early Polytherapy for Probably Benzodiazepine Refractory Naïve Status Epilepticus (Stage 1 Plus)

**DOI:** 10.3390/neurolint17010011

**Published:** 2025-01-19

**Authors:** Giuseppe Magro

**Affiliations:** Department of Neuroscience, “Giovanni Paolo II” Hospital, Lamezia Terme, 88046 Catanzaro, Italy; giuseppemagro.neuro@gmail.com

**Keywords:** stage 1 plus, benzodiazepine refractory status epilepticus, acute etiology in status epilepticus, prolonged status epilepticus, non convulsive status epilepticus, benzodiazepine resistance, early combined polytherapy in status epilepticus, latency to treatment of status epilepticus, treatment delay in status epilepticus

## Abstract

Stage 1 Plus is defined here as a naïve, previously untreated, status epilepticus (SE) that is probably refractory to Benzodiazepines (BDZ). These cases include not only prolonged SE as previously proposed by the author (SE lasting > 10 min) but also other cases notoriously associated with BDZ refractoriness such as the absence of prominent motor phenomena and acute etiology (especially primary central nervous system etiology). Interestingly, the absence of prominent motor phenomena as is the case of non convulsive SE might implicitly fall in the category of prolonged SE due to the delay in recognition and treatment. Future studies should help identify other factors associated with BDZ refractoriness, therefore widening the definition of Stage 1 Plus. The appropriate timing for defining prolonged SE may also differ depending on different etiology. Consequently, in future tailored models of SE, the definition of prolonged SE could be enhanced by defining it for a longer duration than Tx, a time point that changes based on different etiologies (x), Tx being much shorter than 10 min in acute etiologies. These cases of naïve probably BDZ refractory SE (Stage 1 Plus) might require a different approach: combined polytherapy from the start. The objective of this review is to provide pathophysiological and pre-clinical evidence, mostly from animal studies, for the different approach of combined polytherapy from the start for those cases of SE falling in the definition of Stage 1 Plus.

## 1. Introduction

Status epilepticus (SE) is a neurological emergency with an average mortality of 15.9% [1]; nonetheless, delays in treatment are not rare, and happen especially in low-income countries where latency to first-line therapy can be as late as 60 min [2]. Quick cessation of seizure activity is crucial in the treatment of SE. The International League Against Epilepsy has suggested two key time points in SE: T1 and T2. Time point T1 indicates when treatment should be initiated, and time point T2 indicates when long-term consequences may appear. When seizure activity goes on for more than T2, there can be long-term consequences due to neuronal death and altered brain networks [3]. Conventional treatment dictates watching and standing by as first-line therapy with benzodiazepines (BDZ) fails before proceeding to the next step of treatment; as such, stage 2 or Established-SE is defined as BDZ refractory SE, regardless of time [4]. Currently, all guidelines emphasize a step-by-step approach, meaning that second-line therapy is only considered after the failure of first-line treatment. Significant trials, such as the ESETT [5] trial, evaluated second-line agents only after unsuccessful first-line treatment. The American guidelines adopt an escalating strategy. While they take seizure duration into account, it is only about the duration of treatment resistance; thus, stage 2 is classified as a stage that lasts 20 min after the administration of first-line therapy [6] with BDZ. The Italian League Against Epilepsy characterizes stage 2, or definite SE, solely as the “first-line resistant stage” without referencing time in the latest classification. Although early SE has a high response rate within 2 min of BDZ delivery, this rate drops to less than 50% when treatment is delayed 15–30 min [2]. In the study by R. J. Burman et al., “Why won’t it stop? The dynamics of benzodiazepine resistance in status epilepticus”, the authors show a comparison analysis of low-income countries versus high-income countries in terms of latency to treatment; they clearly show that when latency goes to up to 60 min, resistance to first-line BDZ therapy can be as high as 89% in convulsive SE [2]. This delay in treatment translates not only into low response rates but also into long-term consequences. Research involving animals has shown that extended seizures can lead to long-lasting neuronal damage [7]. Serial neuroimaging studies in humans have documented long-term consequences, including cortical atrophy and hippocampal sclerosis, following SE [8]. A longer delay in administering the initial antiseizure medication to stop SE was linked to increased mortality rates and worse functional outcomes [9,10]. This correlation highlights the importance of timely treatment in improving patient prognosis.

Should we then still be treating every SE the same? And most importantly, should physicians from low-income countries that experience this latency in SE treatment every day continue to treat every case the same? A new approach of combined polytherapy from the start is suggested here for those cases of SE falling into the category of Stage 1 Plus: a SE stage, naïve of previous treatment, probably refractory to BDZ, that requires combined polytherapy from the start; as in those cases associated with BDZ resistance such as prolonged seizure activity (≥10 min), acute etiologies, and prevalent non-motor phenomena of SE (Table 1).

The objective of this review is to provide pathophysiological and clinical evidence, mostly from animal studies, for the different approach of combined polytherapy from the start for the cases of SE falling into the definition of Stage 1 Plus.

## 2. Methods and Objectives

A various combination of terms was used for the PubMed search such as “Status Epilepticus” in combination with “treatment delay”, “Benzodiazepine resistance”, and “Benzodiazepine refractory”. The search was conducted until 16 December 2024, with more than 869 records screened by abstract and title and selected in this review based on relevance, especially in providing pathophysiological evidence for combined early polytherapy and identifying factors associated with BDZ resistance. Research papers included were limited to the English language and animal and human adult population. The conclusions drawn in this study are mostly the results of animal studies and pathophysiological studies, with some (but still too few) indirect evidence from observational studies, such as those conducted in low-income countries and comparison analyses. Future studies should help confirm the suggestions provided in this paper.

## 3. Dynamics of Benzodiazepine Resistance in SE

Resistance to BDZ can be predetermined, as in genetic mutations of GABA-A receptors. Still, the most important predictor of resistance to treatment is time itself, and behind time lies the dynamic changes in GABA-A receptor trafficking occurring during prolonged seizure activity. Under normal circumstances, Benzodiazepines bind to Cl^-^ permeable GABA-A receptors and enhance channel conductance, favoring Chloride influx. Since the Chloride concentration is higher in the extracellular space, this results in a hyperpolarizing effect of BDZ. During prolonged seizure activity, many changes occur, the most important ones being the following: GABA-A receptor Cl^-^ gradient inversion (consequent to the failure of active Cl-transport out of the cells, normally mediated by NKCC2), GABA-A receptor internalization, GABA-A receptor trafficking away from synapses, and loss of high BDZ affinity gamma-subunit [11]. This paragraph contains evidence coming from pre-clinical animal models.

### 3.1. Receptor Trafficking

GABA-A receptor trafficking occurs early in SE. GABA-A receptors are heteropentomers primarily assembled with two alpha, two beta, and a gamma or delta subunit [12]. In particular, gamma2 subunit-containing receptors are found at synapses [13], likely due to the high affinity of this subunit with the inhibitory scaffolding protein gephyrin [14]. Conversely, delta subunits lack high affinity for gephyrin and thus are mostly confined to extra-synaptic locations [14]. BDZ sensitivity depends mostly on gamma2 subunits, which bind at the pocket between the alfa–gamma interface [15]. GABA-A receptor subunits are often targets for phosphatase action, and this happens especially with the activity-dependent entry of Ca++ through NMDA receptors or calcium permeant AMPA receptors [16,17] that can lead to protein-phosphatase 2B activation (calcineurin), with dephosphorylation on gamma2 subunits and consequent loss of affinity for gephyrin with consequent lateral diffusion of GABA-A receptors [18]. Loss of GABAergic inhibition in parallel with the development of BDZ resistance occurs early in SE [11], where, as time goes by, Chloride increases its concentration inside the cell; moreover, ongoing seizure activity induces a decrease in the function and surface expression of KCC2, which reduces the Chloride extrusion capacity of neurons [19,20]. This decrease is accompanied by an increase in the expression and activity of NKCC1, which instead favors Chloride influx [21]. In this state, subsequent GABA-A receptors activation can be sufficiently depolarizing to trigger action potentials [22]. Simply put, GABAergic signaling will become excitatory.

All these dynamic changes are time-dependent and are mediated by glutamate over signaling. In several studies using in vivo animal models, increased trafficking with internalization and lateralization away from synapse was observed as soon as 10 min from status epilepticus onset [23,24,25]. Gamma2 subunit containing GABA-A receptor trafficking away from synapses seems determinative for loss of BDZ responsiveness [26]. Paradoxically, GABA-A receptor inversion of the Chloride gradient might shift the GABA signal from classic inhibitory action to an excitatory one. This makes one wonder if a first-line therapy based solely on BDZ current guidelines is an appropriate approach in the setting of prolonged seizures. Moreover, all GABA signaling dysfunction is mediated by NMDA and AMPA receptor over-expression and glutamate over-signaling, which increases as time goes by. The proposed timeline of changes affecting benzodiazepine efficacy during status epilepticus [2] is as follows: as soon as seizure activity starts, Chloride builds up inside the cell, after 10 min from seizure onset, GABA-A receptors become internalized, after 30 min, there is a decreased expression of KCC2 (with a consequent reduction in Chloride extrusion from the cell) and increased expression of NKCC1 (with more influx of Chloride inside the cell), and then GABA-A receptor reconfiguration and extra-synaptic expression happen in later stages.

NMDA receptor trafficking involves N2B receptor subunits increasing, which shows slower deactivation and greater calcium permeability, with potential injury from excitatory currents; moreover, N2B leads to NMDA receptors being expressed at both synaptic and extra-synaptic sites. The increase in N2B subunit-containing receptors, particularly at extra-synaptic sites, can cause depolarizing shifts and hypersynchrony that sustain SE [11]. These changes are all increased by synaptic expression of calcium permeant AMPA receptors during seizure activity [11]. Interestingly, while BDZ lose their efficacy into “late” models of SE, AMPA and NMDA receptor antagonists retain their efficacy late into SE [11]. GABA dysfunction is mediated by NMDA/AMPA and increases with time. Again, this makes one wonder if the absence of any drug targeting AMPA/NMDA in first-line of therapy is still something we can afford in our practice. A combined polytherapy approach for naïve prolonged SE is needed in those cases as many experimental models are showing that targeting AMPA/NMDA and GABA-A receptors at the same time helps to restore unbalanced receptor trafficking [27]. The definition of Stage 1 Plus [28] was suggested by the author for those cases requiring combination therapy from the start, as in the case of prolonged naïve SE (more than 10 min, the time that marks receptor homeostasis disruption).

### 3.2. Other Factors at Play in BDZ Resistance

It is crucial to investigate additional elements that may contribute to the ineffectiveness of first-line treatment; among these elements, neuroinflammation and increased levels of drug-metabolizing enzymes at the blood–brain barrier (BBB) are particularly at play [24,29]. Numerous animal studies have shown that the over-excitation of neural networks during seizure events leads to neurogenic inflammation. Activated microglia and astrocytes at the blood–brain barrier increase the levels of various cytokines and inflammatory mediators, including interleukin-1β (IL1β) and high-mobility group box 1 (HMGB1) [30]. During SE, the levels of IL1β rise and play a role in the resistance seen in extended episodes [31]. The increased activation of the HMGB1 Toll-like receptor 4 pathway is crucial concerning diazepam-resistant SE; particularly, when SE persists for more than 40 min, diazepam alone is insufficient in aborting SE. On the other hand, anti-HMGB1 monoclonal antibodies have shown potential in partially overcoming diazepam resistance in SE animal models [32]. Additionally, it was confirmed in these studies that a combined treatment approach is more effective for prolonged SE episodes. Drug-metabolizing enzymes play a vital role in determining resistance to SE, with P-glycoprotein being especially important. Typically, P-glycoprotein acts as the main efflux transporter for foreign substances across the BBB. However, during instances of SE, there is a significant rise in the expression of P-glycoprotein at BBB, which leads to an increased expulsion of antiseizure medication [29].

## 4. A Role for Etiology Other than Time for BDZ Resistance in SE

Several animal models have shown that resistance to BDZ occurs as SE persists over time, resulting from GABA receptor trafficking [24], as already discussed. Interestingly, even if resistance to BDZ increases with time, not all cases of prolonged SE develop resistance to treatment at the same rate; therefore, other factors must contribute to BDZ resistance, and among these, the most important ones associated with a worse outcome and a lower chance of seizure terminations are an acute etiology, especially primary central nervous system (CNS) disorders, and the absence of motor phenomena during seizures [33], as shown in a prospective human study from Spain. This is also shown in experimental models, where diazepam failed to terminate late SE in animal models of pilocarpine-induced SE but was successful in terminating kainic acid-induced SE [26]. Furthermore, GABA receptor changes were found in the pilocarpine model but not in the kainic acid model [26]. SE model-dependent differences support the hypothesis that the development of BDZ pharmacoresistance may be etiologically predetermined [26].

A logistic regression model adjusted for age, SE semeiology, and consciousness before treatment initiation showed that acute primary CNS, acute secondary CNS, progressive SE, age, and impaired consciousness were significantly associated with increased odds of mortality [34], as shown in a human study from Italy. Potentially fatal etiologies, possibly falling into acute CNS disorders, include the following: acute large vessel ischemic stroke, acute cerebral hemorrhage, acute CNS infections, severe systemic infection, malignant brain tumor, acquired immunodeficiency syndrome with CNS complications, chronic renal insufficiency requiring dialysis, systemic vasculitis, metabolic disturbances, acute intoxication causing coma, eclampsia, and intracranial tumor surgery [35]. This interpretation of acute etiologies falls in the “burden model” of SE, where it was hypothesized that primary CNS insults are accompanied by higher structural damage [4].

As such, the proposed definition of Stage 1 Plus should include not only those cases of prolonged SE (i.e., more than 10 min as previously proposed) but also those cases of probably refractory BDZ SE, and those associated with worse prognosis [34], as in the acute primary CNS etiology setting and absence of prominent motor phenomena, even when not in a prolonged SE case. Interestingly, the absence of prominent motor phenomena as in the case of non convulsive status epilepticus (NCSE) might implicitly fall in the category of prolonged SE due to the delay in recognition and treatment happening more often than in convulsive SE. Whether the association of BDZ refractory SE and the absence of prominent motor phenomena is only the result of delay in recognition and treatment remains to be seen and can be determined in future studies.

## 5. Combined Polytherapy in Naïve SE

**Figure 1:**
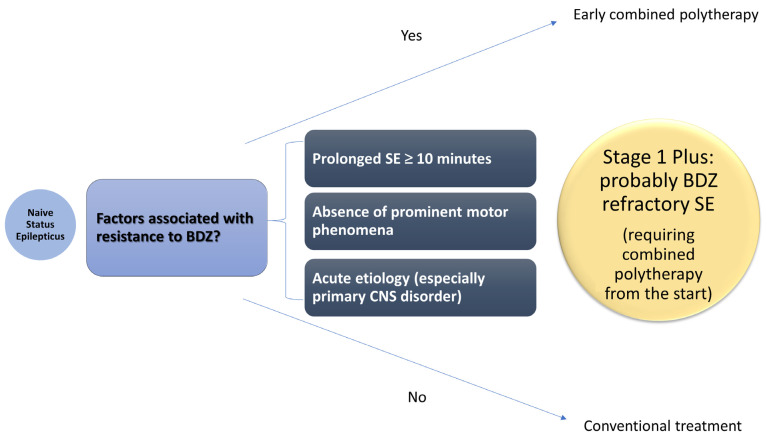
Proposed treatment algorithm for status epilepticus. The presence of factors associated with resistance to treatment with Benzodiazepines (BDZ) in naïve, previously untreated, status epilepticus (SE) defines Stage 1 Plus, a stage requiring a different approach such as combined polytherapy from the start. Interestingly, the absence of prominent motor phenomena as in the case of non convulsive status epilepticus (NCSE) might implicitly fall in the category of prolonged SE due to the delay in recognition and treatment. As of now, acute etiologies (especially primary central nervous system (CNS) disorders) and prolonged SE comprise all the cases of probably BDZ refractory SE. Future studies should help clarify whether the absence of prominent motor phenomena is another BDZ refractory case, independently from latency to treatment, or whether it is just the result of a delay in recognition and treatment. The appropriate timing for defining prolonged SE may also differ depending on different etiology. Consequently, the definition of prolonged SE could be enhanced by considering a longer duration than Tx, a time point that changes based on different etiologies (x), Tx being much shorter than 10 min in acute etiologies. BDZ (benzodiazepines); CSN (central nervous system); SE (status epilepticus).

Episodes of SE resulting from acute brain injuries tend to respond poorly to BDZ, even when administered promptly. This observation suggests that an initial combined therapy approach, incorporating BDZ with antiseizure medications (ASM), could be more effective. Delaying further intervention while awaiting a response to BDZ in such cases may only extend the duration of the SE episode without offering significant benefits. Evidence from animal models of SE induced by acute brain injury supports the effectiveness and rationale of combination therapy [33]. Although some observational studies in clinical settings have reported favorable outcomes, human clinical trials have not yet demonstrated a definitive advantage for this approach [33]. A combined polytherapy from the start, for example, with BDZ plus an antiseizure medication (ASM), might help restore the unresponsive state mediated by AMPA and NMDA receptors in probably BDZ refractory SE (Stage 1 Plus, see Figure 1), making AMPA/NMDA receptor antagonists the ideal choice for the second ASM to accompany BDZ. One could wonder about the reasons for adopting BDZ if there is a high chance of refractoriness; nonetheless, this combination might restore the unresponsive BDZ state being mediated by AMPA/NMDA. Moreover, we still lack the chance of giving up BDZ in current trials as first-line of therapy, as doing so could be considered unethical as of now; future studies should help identify possible new first-line therapies.

Combination therapy should take into account possible side effects of the concomitant multiple drug delivery, and it should be considered with caution in patients with kidney and/or liver disease. However, the combined supra-linear advantages of polytherapy surpass the additive linear toxicities, and administering treatments simultaneously appears to be more effective than doing so sequentially [36]. In simpler terms, drug toxicity was merely additive in experimental models of initial polytherapy [27], showing neither positive nor negative cooperativity.

Combination therapy is more effective when given simultaneously than when given sequentially, as shown in many animal models of both prolonged SE and acute SE etiology [26,27,36,37,38,39].

In studies using animal models, the combination of midazolam and ketamine [37] as a dual therapy has shown to be more effective than either a double dose of midazolam or ketamine alone, as well as valproate combined with midazolam or valproate with ketamine, in alleviating various aspects of SE severity, indicating a synergistic effect. Furthermore, this dual therapy also mitigated acute neuronal damage and the development of epilepsy caused by SE. The findings aligned with the hypothesis that addressing the effects of GABA-A and NMDA receptor trafficking would halt refractory SE.

In the triple simultaneous therapy animal study conducted by Niquet [36] et al., the combination of midazolam, ketamine, and valproate proved to be more effective than a higher dose of dual therapy using midazolam and ketamine in alleviating the severity of SE. This suggests that polytherapy may help reduce the total doses needed for sequential therapy, potentially lowering overall toxicity and decreasing the necessity for escalating to continuous intravenous anesthetic medications, along with its implications for prognosis. The combination of midazolam, ketamine, and valproate effectively stopped the seizures and provided complete neuroprotection in the hippocampus. In contrast, ketamine used alone did not halt the seizures but still offered similar neuroprotection, indicating that injury to hippocampal pyramidal cells is dependent on NMDA [36], therefore, favoring early use of NMAD receptor antagonists. Triple therapies using midazolam, ketamine, and valproate (or phenobarbital) seem to be more effective than dual therapies in decreasing the severity of SE, as indicated by a reduction in EEG signal. Additionally, triple therapies offer enhanced neuroprotection and can help prevent the development of epilepsy, unlike dual or monotherapy in animal models. In line with the receptor trafficking hypothesis, these triple therapies work together to effectively stop acute seizures, which is likely crucial for minimizing or averting their long-term effects [40]. Future studies may assist in pinpointing the polytherapies that optimize the therapeutic index (safety margin of the drugs). The combination of a benzodiazepine (midazolam) to boost GABAergic inhibitory activity with an NMDA receptor blocker (ketamine) that diminishes glutamatergic activity appears to be crucial. Incorporating a third ASM offers further advantages in the cases of valproate and phenobarbital in animal models [40]. Upcoming research should discover additional ASM that can enhance the therapeutic index when paired with midazolam and ketamine while minimizing side effects.

As of today, we lack trials evaluating combined polytherapy from the start in SE based on possible resistance to BDZ. The currently available human trials such as the SAMUKeppra [41] and the Veteran Affairs [42] studies did not select patients for latency to treatment nor acute etiologies, and therefore did not show a clear benefit for this kind of approach. Notably, within the polytherapy group, post hoc analysis revealed a considerable reduction in the proportion of patients experiencing new neurological deficits in the SAMUKeppra trial [41]. Conversely, a study conducted in 2010 assessed the effectiveness of professional practice in comparison to generalized convulsive SE among adults. The findings revealed that the combination of diazepam or clonazepam with fosphenytoin led to a greater rate of seizure termination than using BDZ alone [43]. This suggests that there may be significant advantages associated with the prompt administration of long-acting antiseizure medications other than BDZ. Additionally, the authors highlighted the importance of initiating treatment with long-acting antiseizure drugs at an early stage. One agent that was left out of recent multicenter studies as a second-line treatment alternative is phenobarbital. Low-dose phenobarbital acts as a potent GABA-A receptor agonist, making it susceptible to the same physiological changes in GABA-A receptors that influence the effects of benzodiazepines. However, at elevated doses, phenobarbital appears to be quite effective in halting persistent status epilepticus-like activity in animal models [44]. This effect is linked to pharmacological actions beyond its influence on GABA-A receptors; at higher concentrations, phenobarbital also acts as a potent antagonist of AMPA and kainate glutamatergic receptors [45]. Consequently, phenobarbital may continue to exhibit antiseizure properties, even in brain regions where GABA-A receptor expression was altered or where there is significant intraneuronal Cl^−^ accumulation.

### Low- Versus High-Income Countries

The management and outcome of SE show considerable variations between low- and high-income nations, shaped by disparities in healthcare systems, medication access, and prompt intervention. In low-income environments, late arrival at healthcare facilities and restricted access to advanced diagnostic resources and treatment alternatives, like intravenous benzodiazepines and second-line antiepileptic medications, frequently leads to increased morbidity and mortality rates. Moreover, significant differences in the etiology of SE are observed between low- and high-income countries. For instance, a study on SE in Ethiopian adults identified central nervous system infections as the most prevalent cause [46], thus falling in the category of acute etiology SE. The average rate of resistance to first-line BDZ was found to be higher in studies conducted in low- and middle-income countries compared to those from high-income countries [2]. Therefore, SE in low-income countries might present both the features of prolonged and acute etiology, therefore suggesting that a combined therapy from the start is even more indicated in these countries as Stage 1 Plus is a more frequently encountered scenario.

## 6. Conclusions

Regardless of the cause, all prolonged seizures associated with SE are self-perpetuating and have a low likelihood of stopping on their own. The animal studies already discussed in the previous sections show model-specific variations in the response to diazepam, activity-related trafficking, and synaptic inhibition; giving more credit to the theory that post-synaptic GABA-A receptor population alterations play a role in the emergence of benzodiazepine pharmacoresistance. Additionally, those results support a new hypothesis suggesting that the molecular changes that take place during SE, which may influence treatment response, are also dependent on the underlying cause. A mechanism influenced by both cause and duration could clarify why certain seizures remain treatable with benzodiazepines and other medications, while some causes are more prone to lead to SE episodes that do not respond to treatment. Bringing together all the considerations regarding extended seizure activity and its underlying causes suggest that the appropriate timing for defining prolonged SE may differ depending on different etiologies; besides the already known different times (T1 and T2) based on different seizure semeiology. Consequently, in future tailored models of SE, the definition of prolonged SE could be enhanced by defining it for a longer duration than Tx, a time point that changes based on different etiologies (x), Tx being much shorter than 10 min in acute etiologies, as already discussed. Future studies should help identify the right cut-off time that marks prolonged SE based on different etiologies, especially in NCSE that might implicitly fall in the category of prolonged SE due to the delay in recognition and treatment.

Identifying other factors associated with BDZ resistance might help predict those situations where a different approach is needed. Stage 1 Plus is then meant to include all the cases of naïve refractory status epilepticus that require a different and possibly combined approach from the start.

## Figures and Tables

**Table 1:** Stage 1 Plus definition.

Stage 1 Plus	Conditions Associated with Benzodiazepine Resistance
A stage, naïve of previous treatment, that includes all conditions associated with Benzodiazepine resistance, and that requires combined polytherapy from the start	Prolonged seizure activity (SE lasting ≥ 10 min)
	Acute etiologies (especially primary central nervous system etiologies)
	Prevalent non-motor phenomena of SE

## Data Availability

Not applicable.
